# Monitoring Single DNA Docking Site Activity with Sequential Modes of an Optoplasmonic Whispering-Gallery Mode Biosensor

**DOI:** 10.3390/s25196059

**Published:** 2025-10-02

**Authors:** Narima Eerqing, Ekaterina Zossimova, Sivaraman Subramanian, Hsin-Yu Wu, Frank Vollmer

**Affiliations:** 1 Living Systems Institute, University of Exeter, Exeter EX4 4QD, UK; narima.eerqing@lshtm.ac.uk (N.E.);; 2 Department of Infection Biology, Faculty of Infectious and Tropical Diseases, London School of Hygiene & Tropical Medicine, London WC1E 7HT, UK; 3 Freiburg Center for Interactive Materials and Bioinspired Technologies, Universität Freiburg, 79104 Freiburg, Germany

**Keywords:** single-molecule, plasmonics, whispering-gallery modes, optoplasmonic, multiplexed sensing

## Abstract

In recent years, there has been rapid advancement in single-molecule techniques, driven by their unparalleled precision in studying molecules whose sizes are beyond the diffraction limit. Among these techniques, optoplasmonic whispering gallery mode sensing has demonstrated great potential in label-free single-molecule characterization. It combines the principles of localized surface plasmon resonance (LSPR) and whispering gallery mode (WGM) sensing, offering exceptional sensing capabilities, even at the level of single ions. However, current optoplasmonic WGM sensing operates in a multiplexed channel, making it challenging to focus on individual binding sites of analyte molecules. In this article, we characterize different binding sites of DNA analyte molecules hybridizing to docking strands on the optoplasmonic WGM sensor, using the ratio of the resonance shift between sequential polar WGM modes. We identify specific docking sites that undergo transient interactions and eventually hybridize with the complementary analyte strands permanently.

## 1. Introduction

The examination of life’s processes based on their biomolecular components has been the focus of extensive research spanning several decades. From a physicist’s perspective, these processes typically operate well away from thermodynamic equilibrium [1]. Consequently, predicting the behavior of biomolecules becomes highly challenging due to their existence within complex and non-uniform microbiological systems [2,3,4]. Researchers have employed numerous ensemble averaging techniques to measure the mean state of a biomolecular system. However, even when analyzing a small volume with few biomolecular components, variations can be significant [5]. Relying solely on bulk measurements to determine an average state can result in an inaccurate estimation of individual molecule states within the system and its processes.

In contrast, single-molecule studies offer an unprecedentedly deep and precise understanding of the biology of biomolecular systems and physics of life [6,7]. These studies play a pivotal role in examining the multiple metastable free energy states of biomolecules, with energy states separated by multiples of the thermal energy scale [8,9]. Importantly, this goes beyond the detection capabilities of conventional bulk measurements. Single-molecule techniques facilitate the observation of specific molecular biological features, contributing to the creation of a comprehensive library of molecular heterogeneity within the system. Single-molecule fluorescence is one of the most successful techniques for single-molecule detection [10], relying on the large fluorescence cross-sections of specific organic molecules and proteins. This characteristic enables the detection of fluorescence photons without interference from background signals [11]. Additionally, single-molecule fluorescence resonance energy transfer (FRET) provides valuable insights into the dynamics of biomolecules and chemicals by leveraging distance-dependent energy transfer between donor and acceptor fluorophores attached to a molecule. Due to the requirement for sufficiently high fluorescence quantum efficiencies, fluorophores (labels) must be attached to target molecules in many single-molecule experiments. However, the label often constrains the natural dynamics of the analyte molecule, since it has a similar molecular weight to the analyte molecule [12].

On the other hand, localized surface plasmon resonance (LSPR) sensors represent a single-molecule detection approach free of labels. LSPR sensors normally employ noble metals like gold or silver as the plasmonic nanoparticles. These particles can confine light to nanoscale volumes, creating hotspots where molecules can be detected by local changes in polarizability and refractive index [13,14,15,16]. The optoplasmonic WGM sensing approach offers several advantages over other techniques, such as fluorescence-based methods, optical and magnetic tweezers, and atomic force microscopy (AFM). For example, optoplasmonic WGM sensors have showcased the ability to discern subnanometer-scale conformational changes in active enzymes like MalL with microsecond temporal precision [17]. This real-time monitoring facilitates the observation of enzyme conformational states and enables measurements of thermodynamic parameters such as activation heat capacity.

Monitoring DNA docking site activity at the single-molecule level is crucial for understanding the dynamics of molecular recognition, hybridization kinetics, and binding specificity—core processes underpinning a wide range of biological and biotechnological applications, including gene regulation and DNA-based computation [18,19,20]. The sensitivity of optoplasmonic WGM sensors makes them particularly well-suited for detecting subtle dielectric perturbations induced by molecular interactions at docking sites [13]. Notably, the excess polarizability of single-stranded DNA (ssDNA) influences the local dielectric environment of the sensor, affecting the electromagnetic field distribution and thereby modulating the optical response [21,22]. Variations in these dielectric properties—arising from sequence composition, secondary structure, and hybridization state—contribute to the label-free detection of binding events. When combined with spatially selective sensing strategies, such as mode-resolved resonance analysis, this enables differentiation of individual docking site interactions with high temporal precision.

However, even with these cutting-edge developments mentioned above, it remains challenging to identify and track reactions that happened on each reaction site. In this article, we provide a novel approach capable of identifying and tracing single DNA docking site activity using an optoplasmonic sensor.

## 2. Materials and Methods

### 2.1. Optoplasmonic Sensing of DNA Hybridisation

**Figure 1:**
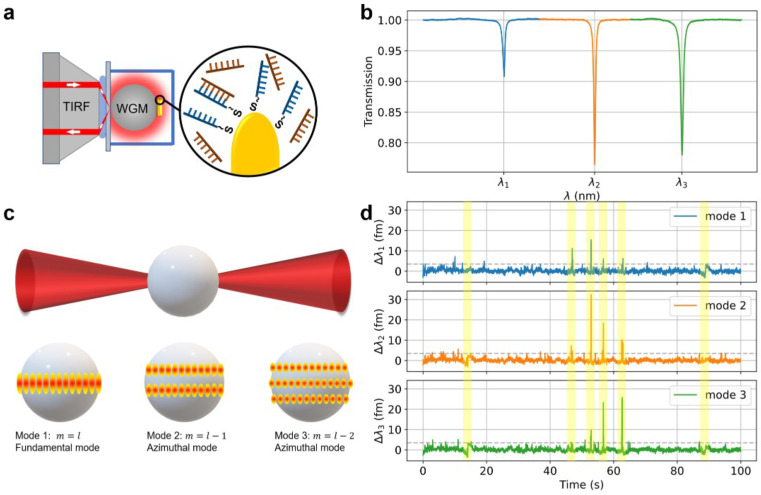
(**a**) Schematic graph of experimental design. An incident laser beam is focused onto the coverslip surface, establishing the total internal reflection. The generated evanescent wave is subsequently coupled into the WGM. The LSPR is excited by the gold nanorods deposited on the surface of the WGM microsphere. (**b**) Transmission spectrum showing the three modes used for the detection of DNA molecules in experiments. (**c**) The schematic graph of the whispering gallery polar modes. Gold nanorods are attached to the WGM sensor and overlap with three adjacent modes corresponding to the mode order. (**d**) Example data-trace of optoplasmonic sensing signals showing the resonance shift, Δλ, experienced by each biosensing mode.

The experiments are carried out on the SIMOPS (single-molecule imaging microscopy and optoplasmonic sensing) [23] platform (Figure 1a). TIRF (Total Internal Reflection Fluorescence) microscopy is utilized to excite evanescent waves, while the external cavity laser scans narrowly around the WGM resonance bandwidth to capture the resonance spectra, and an approximately 80 µm diameter glass sphere is employed to generate WGMs. Gold nanorods (GNRs) are irreversibly immobilized on the glass surface, and LSPR is excited through the WGMs. The docking strands (Figure 1a, zoomed view, shown in blue) are immobilized on the GNRs via a mercaptohexyl linker. Figure 1b illustrates the transmission spectrum of the three sequential modes used in the experiments. Figure 1c depicts the corresponding WGM mode numbers, where the first mode from the left (Figure 1b, blue) represents the fundamental mode, and the others are higher-order modes.

As complementary strands (imagers) approach the docking strands located in the vicinity of the GNRs hotspots, shifts occur in the WGM resonance due to localized perturbations in the refractive index (essentially induced polarization of the molecule by the electric field, leading to a wavelength shift Δλ). Figure 1d displays a snapshot of the wavelength change time trace for each sequential mode in Figure 1b. A centroid fitting algorithm is used to extract the Δλ from the resonance spectra of multiple WGM modes. Custom MATLAB 2019a code is utilized for peak detection, background trend removal (detrending), and signal height measurement. Spike signal detection is performed using a 3σ threshold [13], where σ is the standard deviation of the background signal measured prior to the addition of imagers. Only wavelength shifts exceeding this threshold are recorded, effectively filtering out noise.

**Figure 2:**
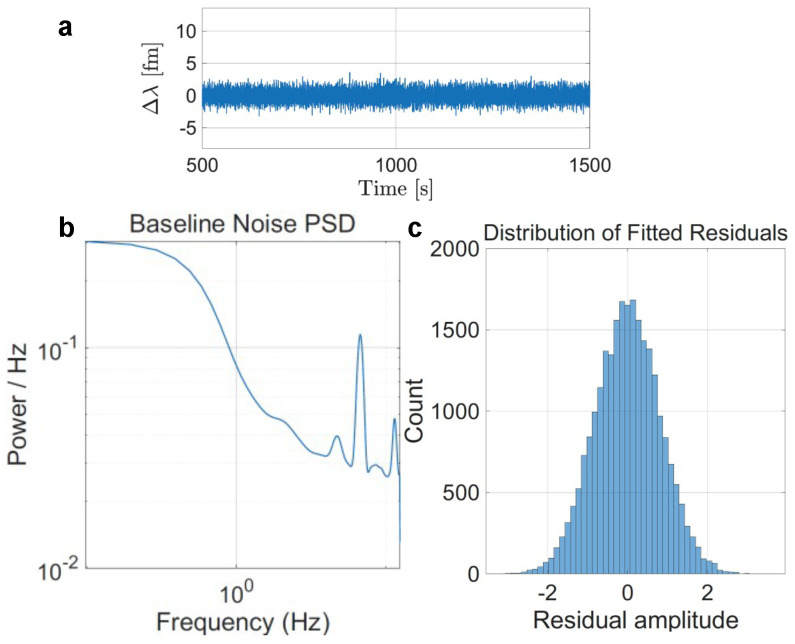
Noise analysis. (**a**) Example of the background trace measuring the sensor immobilized with only docking strands. (**b**) Power spectral density of the baseline noise from a 1000 s background measurement. (**c**) Distribution of fitted residuals obtained from the same 1000 s background trace.

The power spectral density (PSD) of the baseline noise and the distribution of fitted residuals are shown in Figure 2. The PSD generally decreases from low to high frequencies, consistent with slow drift and environmental fluctuations dominating the noise. At higher frequencies, two narrowband peaks are observed, most likely arising from periodic electronic or optical interference (e.g., acquisition electronics or mechanical vibrations) rather than stochastic baseline fluctuations. Their narrowband nature indicates that they are stationary interference components rather than broadband noise, and they were effectively excluded during subsequent thresholding and event detection.

**Figure 3:**
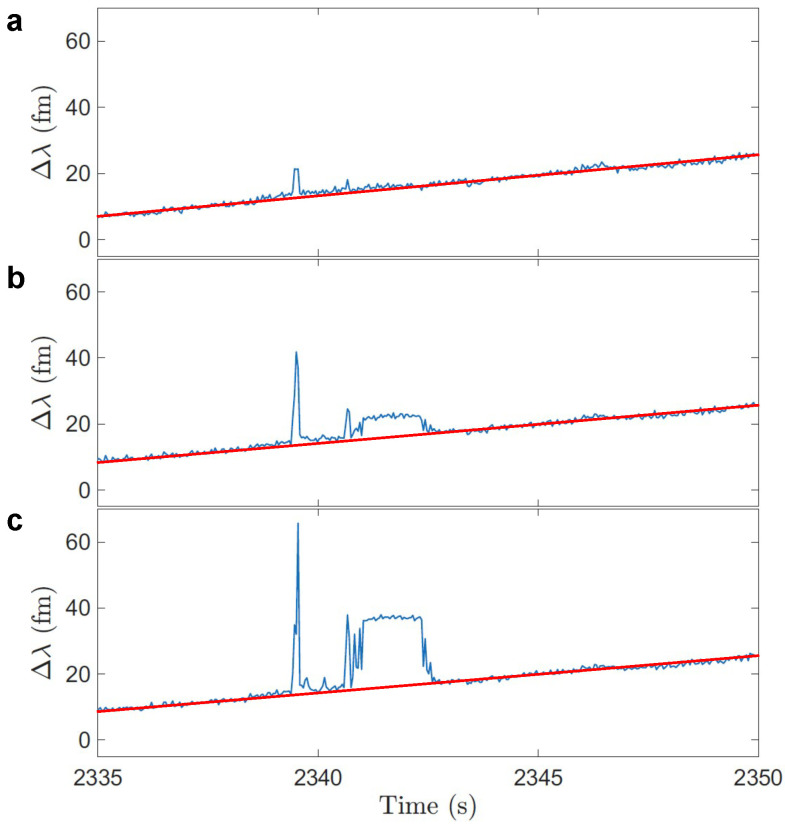
Example trace showing (**a**) Mode 1, (**b**) Mode 2, and (**c**) Mode 3 within the same time frame. A common baseline is applied across all three modes, indicating the absence of mode-dependent baselines.

To test whether mode-dependent baselines affect the RRAs, we applied the same baseline to all three modes. No mode-dependent baseline effects were observed (Figure 3).

### 2.2. Sequential Polar Modes of an Optoplasmonic Resonator

The single-molecule sensing platform comprises a whispering gallery mode (WGM) and several gold nanorods (GNRs). This methodology involves the excitation of two resonances characterized by the same angular momentum quantum number (*ℓ*) but differing magnetic quantum numbers (*m*) within the range of (−*ℓ* < *m* < *ℓ*) in a cavity. While *m* is traditionally known as the magnetic quantum number in atomic physics, when analyzing various *m* modes within a microresonator with a given *ℓ*, the term “polar modes” will be employed. To comprehend the intricacies of this approach, it is essential to scrutinize the polar WGM intensity distribution.

The number of intensity peaks along the polar direction is given by *ℓ* − *m* + 1. In a spherical configuration, these *m* states exhibit degeneracy for a specific angular momentum *ℓ*. However, in geometries where the perfect spherical symmetry of the resonator is broken, such as a spheroid, this degeneracy is resolved, leading to spectral separation of the states. The fundamental mode is an equatorial mode with *m* = *ℓ*, resulting in a single intensity peak centred around the equator. Subsequently, the *m* = *ℓ* − 1 mode displays two peaks, positioned to the North and South of the equator (see Figure 1c). It is crucial to note that these three modes depicted in Figure 1b are activated sequentially within the same microcavity. Similar experiments employing sequential polar modes have been shown by D. Keng et al. [24]. They used a fibre-coupled WGM to detect nanoparticles as they adsorb onto the glass sphere. The ratios of sequential modes were used to determine the latitude angle of where the particles land. In the SIMOPS system, the GNRs are fixed on the WGM microsphere, and single-stranded DNAs perturb the sensor. According to the study by D. Keng et al., the number of shift ratios should be identical to the number of GNR perturbations. However, we observe many more shift ratios than the total number of GNRs, which indicates that there are other factors that cause the various ratios.

Although there are approximately 10 gold nanorods in the experiment, it is difficult to control their exact binding location and orientation on the WGM resonator. Not all of the nanorods will bind to the equator or be oriented at the most optimal angle for maximum field enhancement, which occurs when the long axis of the nanorod is parallel to the polarization direction of the incident light wave [25]. Since the nanorods are scattered randomly across the WGM surface, each plasmonic hot spot will have a unique electric field intensity distribution. In addition, it is well known that surface roughness dramatically affects field enhancement for plasmonic nanoparticles [13,26]. The differences in field intensity across multiple docking sites allow each site to be linked to a unique signal. Any local activity within these sites leads to a small variation in the associated signals, facilitating the identification and analysis of each site.

## 3. Results

### 3.1. Repeating Ratios of WGM Mode Amplitudes Among Adjacent Modes

**Figure 4:**
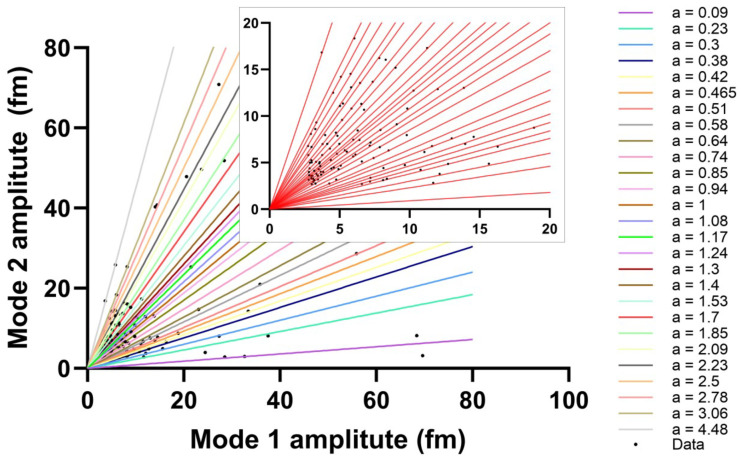
Spike amplitudes of Mode 1 and 2 (black dots) collected from first 45 min of 1 μM ImT22 hybridization experiment. Linear fitting is carried out for the event number greater than 1 (With bin width = 0.02). Corresponding slopes (*a*) are shown and denoted as different colors. Inset figure is the zoomed-in view of Mode 1 and 2 amplitudes within 20 fm. Among all collected data, only 12 data points appeared only once, the remaining 115 data points are shown in repeated slopes.

Spike signal amplitudes for single molecule DNA experiments are collected from a 45-min optoplasmonic sensing experiment with hybridization between 1 μM of ImT22 imager strands (Sequences are shown in Table 1) and complementary docking sites on GNRs. We plot the amplitudes for events that give a signal above the noise threshold for Mode 1 versus Mode 2 in Figure 4.

**Figure 5:**
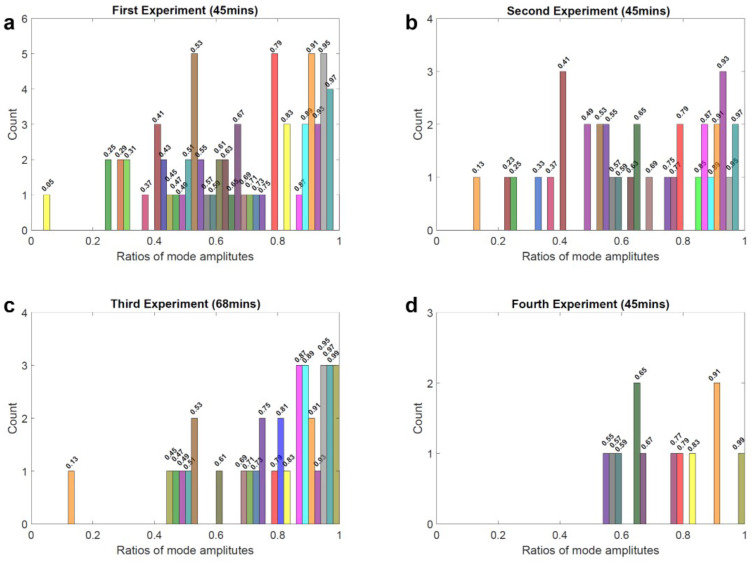
Part of spike amplitude ratios (Ratios between 0 and 1) histogram of Mode 3 and Mode 2 collected from (**a**) first, (**b**) second, (**c**) third and (**d**) fourth continuous optoplasmonic sensing experiments (Bin width = 0.02). The ratios among different experiments are labelled with specific colors.

Further analysis was performed for *m* = *ℓ* − 1 (Mode 2) and *m* = *ℓ* − 2 (Mode 3) across four consecutive experiments, showing consistent results with those observed for Mode 1 and Mode 2. A subset of the ratios is presented as a histogram in Figure 5; only ratios between 0 and 1 are shown, since larger ratios follow the same trend but are more sparsely distributed and therefore omitted for clarity.

Two main characters can be concluded from these four subfigures. Firstly, many RRAs exist across different experiments, some of which show more frequently than others, most likely due to the corresponding docking sites being more accessible. While there are also rare ratios only shown once or twice during experiments over 3 h, suggesting a less accessible docking site. Secondly, there is a decreasing trend in the number of events. The first 45 min of the experiment contain the highest number of events, most ratios gradually disappear over time. In the fourth experiment, only a few ratios remain. This observation matches with what we have reported [23] before; we attributed the reason to the anomalous permanent hybridization between the docking sites and imager strands.

### 3.2. Tracing of Individual Docking Site Interactions

Thus far, we have demonstrated RRAs among the adjacent polar modes (either Mode 2 & Mode 1 or Mode 3 & Mode 2). These ratios illustrate how adjacent polar modes view the same event. One may argue that if these ratios are close to each other, it would be challenging to distinguish them. Here, we present a simple approach to identifying individual sites. If an event is simultaneously captured by all three polar modes, one can obtain two ratios for it: Mode 2 & Mode 1 and Mode 3 & Mode 2. These two ratios are akin to two projections of the same object from different angles. While some objects may appear similar when viewed from one angle alone, they are likely to be different when observed from another perspective.

**Figure 6:**
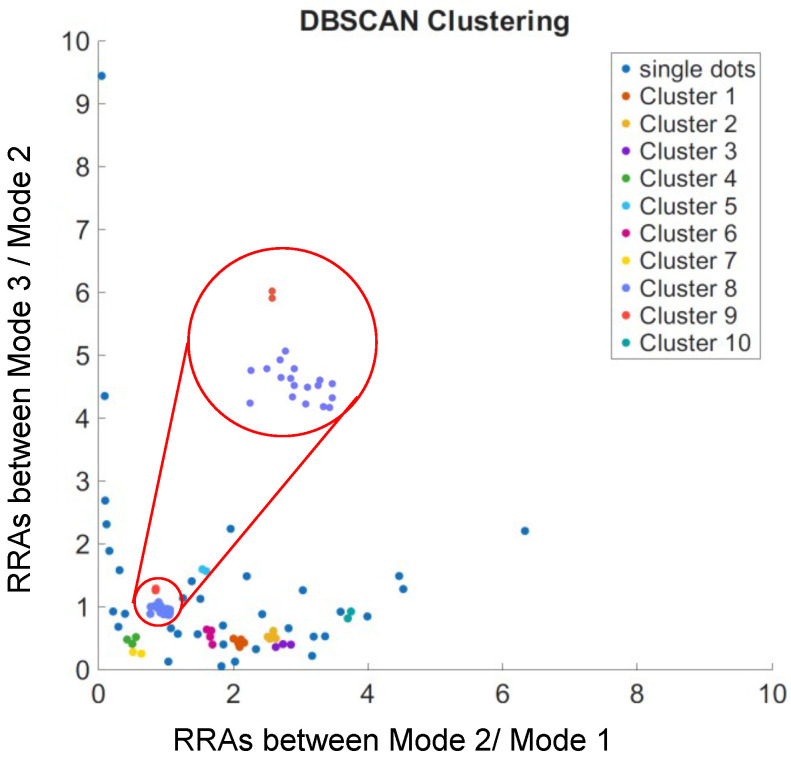
RRAs with similar values form distinct clusters. Red dots in the inset indicate points likely originating from the same docking site, while blue dots represent a larger cluster that may correspond to a more accessible docking site or multiple nearby docking sites.

Figure 6 displays all RRAs from the four experiments, showing several clusters of data points, indicating that those events are likely from the same reaction site. We can easily observe that even though some RRAs share the same ratios between two adjacent modes, they are actually from different sites. We have demonstrated the ability to distinguish single docking sites from multiplexed channels, making it possible to trace the activity from individual docking sites.

We used the DBSCAN clustering method to identify clusters, where neighboring points are grouped based on a distance threshold. To determine the clustering threshold (ϵ) for assigning RRA points to putative single docking sites, we computed the pairwise distances between all RRA points and examined the distribution of nearest-neighbor distances. By selecting ϵ as the 5th percentile of these distances, we captured the typical variation due to measurement noise while avoiding the influence of outliers. This method yielded an empirically optimal ϵ≈0.13, which aligns with the observed grouping of repeated RRA events across experiments. This approach provides a robust and reproducible criterion for identifying site-specific binding events.

### 3.3. Step Signals Correspond to the Disappeared Ratios

For the optoplasmonic sensor, transient interactions are captured in the form of spike signals, where the molecules enter the hotspot of plasmonic nanoparticles and leave shortly after. However, when a binding event occurs, the molecule interacts and stays with the sensor. The polarizability and refractive index within the hotspot are changed, causing a red shift to the WGM spectrum, and therefore observed as a step signal. If the missing ratios correspond to anomalous hybridizations, it is expected that the ratios of the step heights correlate with the ratios of missing spike signals, RRA of spike signal is no longer observed after RRA of step signal indicating permanent hybridisation at single docking site.

**Figure 7:**
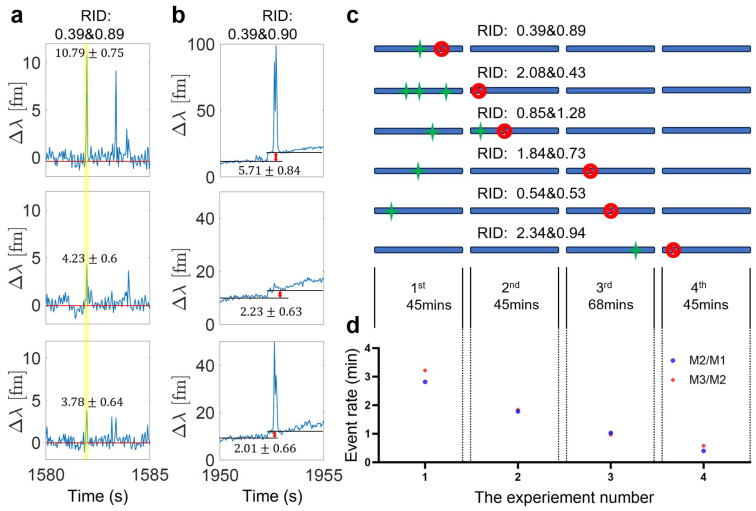
(**a**) Example of a transient interaction (highlighted event) captured by sequential polar modes and their amplitude. The ratio identifier (RRA) denotes the corresponding amplitude ratios between (Mode 2/Mode 1 and Mode 3/Mode 2). (**b**) The step signal with the same RRA is detected after the spike event. (**c**) Spike (green star) and step (red stop sign) signals with the same RRAs captured within four continuous experiments. No spike signals are observed after a step signal, indicating the site is no longer accessible. (**d**) Event rates per minute for the four continuous experiments. Events captured by both Mode 1 and 2 are denoted as blue dots, by both Mode 2 and 3 are denoted as red dots.

We then compared the ratios extracted from the spike signals that showed in the early experiment but then disappeared in the latter experiments with the step signals, in this process, we noticed that some disappeared ratios match with step ratios. We compare RRA step signals to RRA spike signals that occur before the step signal and confirm that these RRAs no longer occur after the step signal. An example of a spike signal (highlighted in yellow) during the first 45 min experiment is shown in Figure 7a; it is detected by three polar modes. Using the measured mode amplitudes, we calculated the Repeating Ratios of Amplitudes (RRA) between adjacent modes. For this spike, the RRA between Mode 1 and Mode 2 was 0.392 ± 0.062, and the RRA between Mode 2 and Mode 3 was 0.894 ± 0.198.

A step signal was observed after approximately 370 s (Figure 7b), and applying the same method yielded similar values, with an RRA of 0.390 ± 0.124 between Mode 1 and Mode 2, and 0.902 ± 0.390 between Mode 2 and Mode 3.

We then examined the following three experiments, and no spikes or steps with similar RRA values were detected.

Similar characterization is conducted and shown in Figure 7c; the green stars denote the time when the spike signal occurs, and the red stop sign corresponds to the time when the step signal appears. We plotted the event rate per minute for the four continuous experiments (Figure 7d), showing events captured by both Mode 1 and Mode 2, as well as Mode 2 and Mode 3. A clear decreasing trend is observed. This result further supports our hypothesis of anomalous hybridization, suggesting that the reduced event rates are caused by the permanent hybridizations between the docking strands and the complementary strands, which blocks the docking sites permanently.

## 4. Discussion

From the wavelength shift time trace, we often observe a shift detected by one or two modes, but no signal is detected by the other mode(s). This is most likely due to the corresponding signal from the other mode(s) falling below the 3σ noise threshold, and only a few modes can be detected by all three modes. This limitation has restricted the capability of employing more WGMs [27], since it is expected that even fewer events can be detected with more modes simultaneously. Therefore, a new method to increase the overall signal-to-noise level is desired to improve data acquisition efficiency.

We have demonstrated the repeating ratios between adjacent polar modes and the approach to identifying and tracing docking sites using the ratios of the resonance shift between sequential polar modes. We have used this method to trace individual docking sites that undergo transient interactions and eventually bond to the docking strands permanently. Evidence shows that the blocked docking sites have lost all of their accessibility, and no further interaction is detected thereafter, matching the observations from previous studies [23].

The RRA approach could become a powerful method to separate signals originating from different sites on many other multiplexed sensing techniques like surface plasmon resonance (SPR) sensing [14], and so on, apart from hybridisation these could be signals originating from enzyme activity, or nanoscale catalysis [28]. It may also be possible to purposely immobilise different docking strands on the sensor and identify the RRA corresponding to a specific sequence in sequential single molecule experiments similar in approach. By using a DNA origami approach [29], for example origami with a nanorod dimer and several different docking sites in the dimer hotspot it may be possible to show that the sensor is well capable of resolving signals from docking sites separated by Angstrom resolution. Taking this idea further, perhaps the sensing signals from proteins labelled with DNA nanobodies allow us to obtain some information on the composition and orientation of a protein aggregate structure on the sensor [30]. A key challenge in applying the RRA method to other platforms such as SPR lies in the requirement for detecting single-molecule signals and the ability to read out these signals across multiple channels. While this level of sensitivity and multiplexing exceeds the capabilities of most conventional SPR sensors, recent reports in the literature suggest that single-molecule detection using SPR is becoming feasible, and developments in multiplexed SPR readouts are also emerging [31]. These advances indicate that, although technically challenging, the extension of the RRA method to SPR-based platforms may be achievable with further technological progress.

The development of computational models can complement experimental studies by providing additional insights into the electronic response properties of target molecules. Recent advances in this area, as reported by Cammi [22] and Booth [32], have enabled the calculation of excess polarizabilities of target molecules, with applications in optoplasmonic sensing. These calculated values can be correlated with the observed wavelength shifts (Δλ) in optoplasmonic data traces [33]. However, accurately accounting for the nanoscale field gradients induced by plasmonic nanoparticles remains challenging, due to heterogeneity in their binding location and orientation on the optoplasmonic resonator [34]. Closer integration of computational and experimental approaches could ultimately improve the interpretation of optoplasmonic sensing signals and enhance molecular-level understanding.

## Figures and Tables

**Table 1:** Sequences of ssDNA used for the experiments.

	ssDNA	Sequence (5′-3′)
Set I	P1	[ThiolC6] TTT TAT ACA TCT A
	ImP1*D	[DY782] CTA GAT GTA T
Set II	T22	[ThiolC6] TTT TGA GAT AAA CGA GAA GGA TTG AT
	ImT22*D	[DY782] ATC AGT CCT TTT CCT TTA TCT C (3 mismatched)

## Data Availability

The data presented in this study are available from the authors upon reasonable request.

## References

[1] Fang X., Kruse K., Lu T., Wang J. (2019). Nonequilibrium physics in biology. Rev. Mod. Phys..

[2] Lenn T., Leake M.C. (2012). Experimental approaches for addressing fundamental biological questions in living, functioning cells with single molecule precision. Open Biol..

[3] Alberts B., Barry J., Bedinger P., Formosa T., Jongeneel C., Kreuzer K. (1983). Studies on DNA Replication in the Bacteriophage T4 a–.gif System. Cold Spring Harb. Symp. Quant. Biol..

[4] Nossal N.G., Makhov A.M., Chastain P.D., Jones C.E., Griffith J.D. (2007). Architecture of the bacteriophage T4 replication complex revealed with nanoscale biopointers. J. Biol. Chem..

[5] Miller H., Zhou Z., Shepherd J., Wollman A.J.M., Leake M.C. (2017). Single-molecule techniques in biophysics: A review of the progress in methods and applications. Rep. Prog. Phys..

[6] Capitanio M., Pavone F.S. (2013). Interrogating biology with force: Single molecule high-resolution measurements with optical tweezers. Biophys. J..

[7] Yu B., Chen D., Qu J., Niu H. (2011). Fast Fourier domain localization algorithm of a single molecule with nanometer precision. Opt. Lett..

[8] Kufer S.K., Strackharn M., Stahl S.W., Gumpp H., Puchner E.M., Gaub H.E. (2009). Optically monitoring the mechanical assembly of single molecules. Nat. Nanotechnol..

[9] Hinterdorfer P., Dufrêne Y.F. (2006). Detection and localization of single molecular recognition events using atomic force microscopy. Nat. Methods.

[10] Weiss S. (1999). Fluorescence spectroscopy of single biomolecules. Science.

[11] Reinhardt S.C.M., Masullo L.A., Baudrexel I., Steen P.R., Kowalewski R., Eklund A.S., Strauss S., Unterauer E.M., Schlichthaerle T., Strauss M.T. (2023). Ångström-resolution fluorescence microscopy. Nature.

[12] Deniz A.A., Mukhopadhyay S., Lemke E.A. (2008). Single-molecule biophysics: At the interface of biology, physics and chemistry. J. R. Soc. Interface.

[13] Baaske M.D., Foreman M.R., Vollmer F. (2014). Single-molecule nucleic acid interactions monitored on a label-free microcavity biosensor platform. Nat. Nanotechnol..

[14] Baaske M.D., Asgari N., Punj D., Orrit M. (2022). Nanosecond time scale transient optoplasmonic detection of single proteins. Sci. Adv..

[15] Lin Y.T., Vermaas R., Yan J., de Jong A.M., Prins M.W. (2021). Click-coupling to Electrostatically Grafted polymers greatly improves the stability of a continuous monitoring sensor with single-molecule resolution. ACS Sens..

[16] Seth A., Mittal E., Luan J., Kolla S., Mazer M.B., Joshi H., Gupta R., Rathi P., Wang Z., Morrissey J.J. (2022). High-resolution imaging of protein secretion at the single-cell level using plasmon-enhanced FluoroDOT assay. Cell Rep. Methods.

[17] Subramanian S., Jones H.B., Frustaci S., Winter S., van der Kamp M.W., Arcus V.L., Pudney C.R., Vollmer F. (2021). Sensing Enzyme Activation Heat Capacity at the Single-Molecule Level Using Gold-Nanorod-Based Optical Whispering Gallery Modes. ACS Appl. Nano Mater..

[18] Zhang D.Y., Winfree E. (2009). Engineering entropy-driven reactions and networks catalyzed by DNA. Science.

[19] Yurke B., Turberfield A.J., Mills A.P., Simmel F.C., Neumann J.L. (2000). A DNA-fuelled molecular machine made of DNA. Nature.

[20] Seeman N.C. (2003). DNA in a material world. Nature.

[21] Petersen C., Heimburg T., Mouritsen O.G., Zuckermann M.J. (2015). Dielectric response of single-stranded DNA: An experimental and theoretical study. J. Phys. Chem. A.

[22] Cammi R. (2025). The Excess Polarizability of Single-Stranded DNA Molecules in Solution: A Linear Response Theory in the Polarizable Continuum Model with an Application to Biosensing. J. Phys. Chem. A.

[23] Eerqing N., Wu H.Y., Subramanian S., Vincent S., Vollmer F. (2023). Anomalous DNA hybridisation kinetics on gold nanorods revealed via a dual single-molecule imaging and optoplasmonic sensing platform. Nanoscale Horizons.

[24] Keng D., Tan X., Arnold S. (2014). Whispering gallery micro-global positioning system for nanoparticle sizing in real time. Appl. Phys. Lett..

[25] Foreman M.R., Vollmer F. (2013). Theory of resonance shifts of whispering gallery modes by arbitrary plasmonic nanoparticles. New J. Phys..

[26] Novotny L., Hecht B. (2012). Principles of Nano-Optics.

[27] Vollmer F., Arnold S. (2008). Whispering-gallery-mode biosensing: Label-free detection down to single molecules. Nat. Methods.

[28] Houghton M.C., Toropov N.A., Yu D., Bagby S., Vollmer F. (2024). Single molecule thermodynamic penalties applied to enzymes by whispering gallery mode biosensors. Adv. Sci..

[29] Rothemund P.W. (2006). Folding DNA to create nanoscale shapes and patterns. Nature.

[30] Jäger M., Nir E., Weiss S. (2006). Site-specific labeling of proteins for single-molecule FRET by combining chemical and enzymatic modification. Protein Sci..

[31] Liu T., Hu S., Ding S., Zhang L., Yi C., Chen Y., Liu G.S., Chen Z., Xiao W., Chen L. (2025). Algorithm assisted comprehensive optimization of SPR sensors towards single molecule detection. Biosens. Bioelectron..

[32] Booth L.S., Browne E.V., Mauranyapin N.P., Madsen L.S., Barfoot S., Mark A., Bowen W.P. (2022). Modelling of the dynamic polarizability of macromolecules for single-molecule optical biosensing. Sci. Rep..

[33] Vollmer F., Yu D. (2022). Optical Whispering Gallery Modes for Biosensing: From Physical Principles to Applications.

[34] Zossimova E., Jones C., Perera K.M.K., Pedireddy S., Walter M., Vollmer F. (2024). Whispering Gallery Mode Sensing through the Lens of Quantum Optics, Artificial Intelligence, and Nanoscale Catalysis. Appl. Phys. Lett..

